# Accelerated 4D flow imaging with variable-density cartesian undersampling and parallel imaging reconstruction

**DOI:** 10.1186/1532-429X-15-S1-P11

**Published:** 2013-01-30

**Authors:** Jing Liu, Petter Dyverfeldt, Michael D Hope, David Saloner

**Affiliations:** 1 Radiology and Biomedical Imaging, University of California San Francisco, San Francisco, CA, USA; 2 Linköping University Hospital, Linköping, Sweden

## Background

4D flow CMR is hampered by long scan times. We investigated an effective undersampling scheme and an efficient parallel image reconstruction method to achieve highly accelerated 4D flow CMR with high reconstruction accuracy.

## Methods

**Figure 1:**
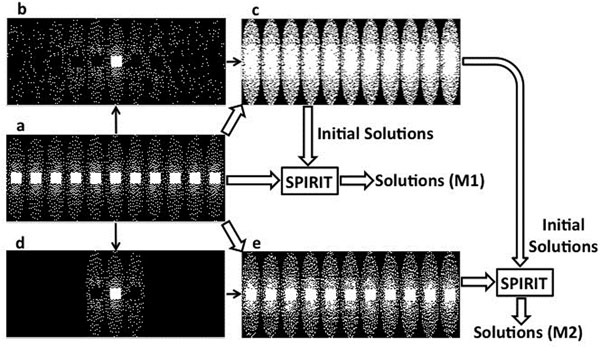
a) Variable-density Poisson-Disk Distribution sampling on ky-kz plane through time (each block is for one time frame; k-space center is fully sampled), b) the selected samples at each time frame for generating a composite data used for the middle frame, c) composite sampling patterns through time for generating initial solutions for SPIRIT, d) the selected samples for generating a composite image for the middle frame, e) new composite data with reduced undersampling.

Variable-density Poisson Disk Distribution (VD-PDD) undersampling was applied for 4D flow imaging. By applying VD-PDD independently at each time frame (Figure 1a), we achieved random undersampling in both the ky-kz plane and temporal domains. In addition, we applied an improved initial solution for SPIRIT to significantly improve reconstruction accuracy and robustness.

We explored the different undersampling and reconstruction algorithms on fully sampled 4D flow CMR data acquired on a 1.5T Siemens Avanto scanner with a 5-ch coil in 3 volunteers (venc=200 cm/s, FOV=320x240x55 mm^3^, matrix=128x96x22, ~18 time frames of 35 ms temporal resolution, ~25 mins scan time). VD-PDD (center 12x12 fully sampled; R=6) was retrospectively applied to the full data.

**Figure 2:**
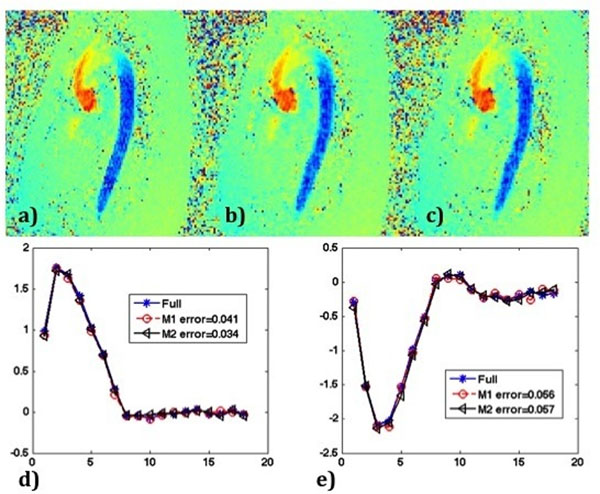
Flow images obtained with a) R=1 (full), b) R=6 VD-PDD and SPIRIT with improved initial solution (M1), and c) R=6 VD-PDD with reduced undersampling and SPIRIT with improved initial solution (M2). d-e) show representative flow-waveforms of AA and DA with full data, M1 and M2.

Composite data was generated by sharing data from other frames based on the temporal distance to the time frame of interest (Figure 1b). k-space was filled to the extent possible from selected adjacent time frames. The images reconstructed from the composite data through time (Figure 1c) were used as initial solutions for SPIRIT. This is denoted as method "M1". We also generated composite data that shared limited data from other frames (Figure 1d) to mitigate undersampling of the data. Then SPIRIT with the proposed initial solutions was applied to this new composite data through time (Figure 1e), referred to as "M2". Flow-waveforms in the ascending (AA) and descending aorta (DA) were measured in 5 locations in each subject (Figure 2d-e). Relative error was calculated with the fully sampled data as reference.

## Results

Both reconstruction methods were successfully applied to all subjects. Velocity images compared favorably to the fully sampled velocity image (Figure 2a-c). The relative error in flow measurement was 0.05±0.01 (AA, M1), 0.04±0.01 (AA, M2), 0.09±0.05 (DA, M1), and 0.07±0.02 (DA, M2). Both proposed methods achieved outstanding performance with 6-fold acceleration. Of the two, M2 had smaller errors, indicating that an effective temporal sharing scheme combined with VD-PDD and SPIRIT could be a potential way of improving image quality without sacrificing temporal resolution.

## Conclusions

We employed undersampling patterns based on VD-PDD, parallel imaging method SPIRIT, and a temporal sharing scheme to achieve 6-fold accelerated 4D flow CMR with a small number of coils. The qualitative and quantitative comparisons indicate the potential of our methods to achieve highly accelerated flow imaging with maintained accuracy. Future work includes implementation of prospective undersampling.

## Funding

American Heart Association BGIA

NIH NIBIB K25
